# Clustering of developmental delays in Bavarian preschool children – a repeated cross-sectional survey over a period of 12 years

**DOI:** 10.1186/1471-2431-14-18

**Published:** 2014-01-23

**Authors:** Heribert L Stich, Alexander Krämer, Rafael T Mikolajczyk

**Affiliations:** 1 Department of Public Health Medicine, District of Erding, Erding 85435, Germany; 2 Department of Public Health Medicine, School of Public Health, University of Bielefeld, Bielefeld 33501, Germany; 3 Department of Epidemiology, Helmholtz Centre for Infection Research, Braunschweig, Germany; 4 Hannover Medical School, Hannover, Germany

**Keywords:** Developmental delays, Distribution pattern, Preschool children, Preventive medicine

## Abstract

**Background:**

While most children display a normal development, some children experience developmental delays compared to age specific development milestones assessed during school entry examination. Data exist on prevalence of delays in single areas, but there is lack of knowledge regarding the clustering patterns of developmental delays and their determinants.

**Methods:**

During the observation period 1997-2008, 12 399 preschool children (5-7 years of age) in one district of Bavaria, Germany, were assessed in twelve schooling-relevant development areas. The co-occurrence of developmental delays was studied by means of Pearson’s correlation. Subsequently, a two-step cluster algorithm was applied to identify patterns of developmental delays, and multinomial logistic regression was conducted to identify variables associated with the specific patterns.

**Results:**

Fourteen percent of preschool children displayed developmental delays in one and 19% in two or more of the studied areas. Among those with at least two developmental delays, most common was the combination of delays in "fine motor skills" + "grapho-motor coordination" (in 9.1% of all children), followed by "memory/concentration" + "endurance" (5.8%) and "abstraction" + "visual perception" (2.1%). In the cluster analysis, five distinct patterns of delays were identified, which displayed different associations with male gender and younger age.

**Conclusions:**

While developmental delays can affect single areas, clustering of multiple developmental delays is common. Such clustering should be taken into account when developing diagnostic tests, in pediatric practice and considering interventions to reduce delays.

## Background

In the international comparison, developmental delays are defined and assessed differently across countries [1-3]. This is not only the case for single developmental delays, but even more for the co-occurrence of delays. In Germany, the term "performance deficits" was defined with focus on relevant skills for entering 1^st^ class of primary school [3]. In this definition only the occurrence of single development delays was recognized [3]. In contrast, in the U.S.A. and in Canada primarily specific combinations of developmental disabilities were in the focus of interest. The term "development disability" was used for developmental delays which manifested before the 18^th^ birthday and affected daily functioning in three or more of the following areas: capacity for independent living, economic self-sufficiency, learning, mobility, language receptive and expressive, self-care, and self-direction [4]. Thus, it is not surprising that in the Anglo-Saxon countries the incidence of combined developmental delays received more attention in the relevant literature than in German-speaking countries.

The acquisition of various skills in the context of individual development is a very differentiated process and varies from child to child [5,6]. Although the vast majority of children in the Western industrialized countries experience an intact somatic, psychological and social development, a variety of developmental trajectories can be observed and developmental delays can be identified [1,7]. Most of previous studies considered delays independently of each other, with focus on motor and language development on the one side [2], and cognitive or mental delays in specific patient populations on the other side [8,9]. In the diagnostic practice for a non-negligible number of preschool children not only a single, isolated developmental delay, but a clustering of delays can be observed. Publications in the area of combined developmental delays appeared first in the early 1990s. Bishop [10] and Nicholoson and Fawcett [11] noticed a combination of delays in development of coordination and language disorders in children. According to other authors, language disorders frequently were associated with attention disorders [9,12,13] or with abnormalities in motor skills, attention and psychosocial development [3,14,15]. While the observations of co-occurrence of developmental delays were made, no exact frequencies were reported. This fact represents a considerable deficit, because it is known that especially combined developmental delays usually have moderate or strong expression, while isolated delays have rather a mild expression [16]. Further, combined developmental delays have a tendency to persistence [17]. The knowledge of these facts might be important for diagnostics in the field of childhood development.

The present study aimed to assess the co-occurrence of developmental delays using data from a school entry examination, which is mandatory for preschool children in Germany and therefore provides an unselected population-based, non-clinical sample. Prevalences of single developmental delays were subject to previous analyses in the same District of Bavaria [18,19].

## Methods

### Study design

The present investigation is based on a repeated survey using the framework of the mandatory school entry examination in Germany and therefore including each year the complete age cohort at about 5-6 years [20]. In the presence of a severe disease of the eyes or hearing, the child was not considered for standardized school entry examination and not included in the sample. We included in the analysis all children participating in school entry examinations in the years 1997-2008 who had primary residence in the Lower Bavarian District Dingolfing-Landau (N = 12 399).

### Content of the examination

In the study district, a manual of the Working Group "School and Youth Health Care in the Public Health Service" was used in a slightly modified form [21]. The corresponding test battery was designed to assess four dimensions of development with corresponding subareas - in total 12 developmental areas (Table 1). All tests used in the diagnostics are standardized, and every child had to absolve the complete examination. If a test could not be performed as requested, this was considered as a developmental delay in the corresponding subarea.

**Table 1:** Modification of "Bavarian School Entry Model" used in the current study for the assessment of developmental delays

**Assessment**	**Main areas of development**	**Subareas**	**Standardized investigation procedures**
Biomedical	Motor	Gross motor	Standing on one leg (at least 10 s, both legs, max. 3 trials), jumping on one leg (at least 5 times on each leg), walking like a rope dancer (20 steps forwards and backwards), walking and clasping hands (walk a 10 m walk a 10 m walk clasping hands at each step)
		Fine body coordination	Finger-opposition-test (touching with the thumb all other fingers from 2 to five and backwards, max. 3-4 s pro sequence, per hand), fist-palm-test (one hand clenched to fist the other as palm and change of hands 7-10 times in 10 s), thumb-palm-test (as previous one but with the thumb and palm)
		Grapho-motor coordination	Painting of a human figure (head with eyes, mouth, ears, hairs, body and hands and legs), tracing of geometric shapes (four shapes: circle, cross, triangle, square), colouring of objects (colouring should stay within shapes), drawing of curved lines (line should stay within a curved 15 cm long 1 cm wide area), connecting points with a straight line (two points in 15 cm distance should be connected by a straight line)
	Language	Pronunciation	Repeating words (8-10 words with specific consonants and vocals have to be repeated), repeating simple sentences (7 defined sentence with increasing difficulties); repeating nonsense-words (7 defined non-sense words with specific consonants and vocals) (one misspelling is acceptable)
		Grammar	Retelling a short story (5 sentences), explaining rules of a known game (for example football)
		Rhythm of speech	Repeating of longer sentences with specific sounds
	Cognition	Memory & concentration	Repeating sentences with 7-10 words including 3 adjectives; repeating 4 single numbers in a correct sequence
		Endurance	Capacity to attend during the examination (15-20 minutes)
		Abstraction	Building pairs (14 pictures with household goods), finding a common subject of various objects, finding difference between pictures
		Visual perception	Recognition of simple geometric figures or silhouettes of figures and animals
		Arithmetics	Counting from 1 to 10 in correct sequence
Psychological	Psychosocial		Erratic behaviour, overly bonded mother (no separation possible during examination), hostility towards examiner
			Major emotional mood
			Psycho-motor agitation, inability to sit calmly during examination

Note: There is some overlap between tests for fine motor and grapho-motor development, requiring interpretation by the attending expert.

Diagnosis and documentation of findings were performed by the investigation team of the School Health Service in the district of Dingolfing- Landau. During the entire twelve years study period, this medical team was composed of the same personnel and used the same approach. The analysis is based on anonymized data obtained in these routine examinations and was approved by the ethics committee of the University of Bremen.

### Statistical analyses

For data analysis, software package SPSS 19.0 was used [22]. First, we performed descriptive analyses of the sample. Second, in order to assess if some delays are more often combined with others, we used Pearson’s correlation coefficient. Given the large sample size, even marginal correlations were significant. Therefore, instead of using significance criterion, we used Cohen’s classification of effect sizes for interpretation and focused only on correlations of 0.5 or higher which are considered strong [23]. Next, among children with at least one delay, we studied the clustering of delays beyond just a combination of two delays by means of a two-step cluster algorithm [24]. Finally, we used multinomial logistic regression analysis to identify variables independently associated with specific patterns of delays, considering children with "no delays" as the reference group.

## Results

### Sociodemographic characteristics

The District of Dingolfing-Landau has over 91,000 inhabitants. The area has a rural infrastructure besides one industrial factory of automobiles. About 93% of the population has the German nationality. Average age of the examined children was 5.95 years (standard deviation 0.39); 51.7% of all children were male, and 89.5% had the German nationality.

### Description of prevalence of delays

Of all 12,399 preschool children examined during the study period, approximately two-thirds did not demonstrate any delays, 14.2% had one, 6.8% had two, and the remaining 11.6% - three or more developmental delays. Highest co-occurrences of delays were found for *fine body coordination* and *grapho-motor coordination* (9.1%) and for *memory/concentration* and *endurance* (5.8%) (Table 2).

**Table 2:** Prevalence of combinations of developmental delays (cp = in percent) and corresponding correlations (r = Pearson’s correlations coefficients)

	**Gross motor**	**Fine body coordination**	**Grapho-motor coordination**	**Pronunciation**	**Grammar**	**Rhythm of speech**	**Memory & concentration**	**Endurance**	**Abstraction**	**Visual perception**	**Arithmetic**	**Psychosocial**
Gross motor		r = 0.38	r = 0.30	r = 0.11	r = 0.13	r = 0.09	r = 0.30	r = 0.27	r = 0.19	r = 0.21	r = 0.20	r = 0.23
Fine body coordination	cp = 3.5%		**r = 0.78**	r = 0.13	r = 0.16	r = 0.09	r = 0.36	r = 0.32	r = 0.19	r = 0.20	r = 0.25	r = 0.23
Grapho-motor coordination	cp = 2.6%	**cp = 9.1%**		r = 0.12	r = 0.17	r = 0.09	r = 0.33	r = 0.33	r = 0.20	r = 0.20	r = 0.25	r = 0.22
Pronunciation	cp = 1.8%	cp = 3.0%	cp = 2.4%		r = 0.23	r = 0.10	r = 0.12	r = 0.11	r = 0.07	r = 0.08	r = 0.08	r = 0.09
Grammar	cp = 0.8%	cp = 1.4%	cp = 1.2%	cp = 2.0%		r = 0.19	r = 0.22	r = 0.19	r = 0.13	r = 0.13	r = 0.12	r = 0.13
Rhythm of speech	cp = 0.6%	cp = 0.8%	cp = 0.7%	cp = 1.0%	cp = 0.8%		r = 0.10	r = 0.06	r = 0.05	r = 0.07	r = 0.06	r = 0.06
Memory and concentration	cp = 2.9%	cp = 4.9%	cp = 3.9%	cp = 2.8%	cp = 1.7%	cp = 0.9%		**r = 0.66**	r = 0.26	r = 0.29	r = 0.35	r = 0.35
Endurance	cp = 2.0%	cp = 3.3%	cp = 2.9	cp = 1.9%	cp = 1.2%	cp = 0.5%	**cp = 5.8%**		r = 0.27	r = 0.26	r = 0.32	r = 0.38
Abstraction	cp = 1.0%	cp = 1.4%	cp = 1.3%	cp = 0.9%	cp = 0.6%	cp = 0.3%	cp = 1.8%	cp = 1.4%		**r = 0.54**	r = 0.27	r = 0.23
Visual perception	cp = 1.3%	cp = 1.8%	cp = 1.5%	cp = 1.2%	cp = 0.6%	cp = 0.4%	cp = 2.3%	cp = 1.6%	**cp = 2.1%**		r = 0.27	r = 0.19
Arithmetic	cp = 1.2%	cp = 2.0%	cp = 1.7%	cp = 1.1%	cp = 0.6%	cp = 0.3%	cp = 2.5%	cp = 1.8%	cp = 1.1%	cp = 1.2%		r = 0.20
Psychosocial	cp = 1.7%	cp = 2.6%	cp = 2.1%	cp = 1.6%	cp = 0.8%	cp = 0.5%	cp = 3.4%	cp = 1.4%	cp = 1.2%	cp = 1.2%	cp = 1.2%	

Note: Correlations above 0.5 are marked in bold. All correlations were highly significant (p < 0.0001).

### Clustering of developmental delays

#### Correlations of developmental delays in different areas

Developmental delays of *fine motor coordination* and *grapho-motor coordination* showed the strongest correlation (Pearson’s correlation coefficient r = 0.78, Table 2). Also, a high correlation was found between developmental delays in the subareas of *memory/concentration* and *endurance* (r = 0.66), and in the subareas of *capacity for abstract thinking* and *visual perception* (r = 0.54, Table 2). The remaining correlation coefficients were below 0.5. Despite the differences in strength, all correlations were highly significant (p < 0.0001).

#### Patterns of concurrent delays

In the cluster analysis restricted to children with a least one developmental delay, five distinct patterns of developmental delays were identified (Table 3). We described the patterns based on most frequent areas of impairment in the corresponding pattern using the following algorithm: first, all those which were recorded in at least 50% of cases; second, if there was only one area above 50%, a second area with high ratings was included; and third, if multiple delays to be included in the definition differed by less than 5%, they were all included in the description of the given pattern. The first pattern were isolated disorders in *pronunciation of speech*, the second pattern - combined delays in subareas of *pronunciation, grammar, rhythm of speech and psychosocial development,* the third pattern - deficits of subareas of *memory/concentration, endurance, abstraction* and *visual perception*. The fourth pattern was dominated by delays of *fine body coordination* and *grapho-motor coordination*. The fifth pattern was a combination of cognitive and motor developmental delays (*fine motor coordination, grapho-motor coordination, memory/concentration, endurance*). In the patterns three to five, delays in some further areas had also high prevalence.

**Table 3:** Patterns of developmental delays among preschool children with at least one delay in individual development*

**Subareas of development**	**Pattern 1**	**Pattern 2**	**Pattern 3**	**Pattern 4**	**Pattern 5**
	**Isolated disorders of pronunciation**	**Combined delays of pronunciation, grammar, rhythm of speech and psychosocial development**	**Delays in development of memory, concentration, endurance, abstraction and visual perception**	**Delays of fine body coordination and grapho-motor coordination**	**Combination of delays in cognitive and motor development**
Gross motor	0%	23%	0.12	21%	41%
Fine body coordination	0%	0%	0%	**93%**	**94%**
Grapho-motor coordination	0%	0%	0%	**68%**	**77%**
Pronunciation	**100%**	**30%**	21%	22	30%
Grammar	0%	**29%**	8%	4%	21%
Rhythm of speech	0%	**34%**	3%	5%	10%
Memory and concentration	0%	6%	**69%**	0%	**88%**
Endurance	0%	1%	**39%**	1%	**58%**
Abstraction	0%	0%	21%	0%	26%
Visual perception	0%	1%	31%	3%	29%
Arithmetic	0%	1%	23%	4%	30%
Psychosocial	0%	**28%**	27%	11%	33%

*Presented are the percentages of children presenting delays among those identified as members of the specific cluster; the dominating delays for each cluster are marked in bold and used for the description of the cluster.

#### Variables associated with patterns of delays

Compared to children without any delays, male and younger children had a higher risk for any combination of delays (Table 4). The effects of both factors were less pronounced for disorders of language development (Pattern 1 or 2), while they were substantially stronger for combinations with delays in fine body coordination (Pattern 4) and motor development (Pattern 5). Migration background was associated with a lower risk for isolated delays of pronunciation (Pattern 1) and a higher risk for all other patterns (Pattern 2 to 5) (Table 4).

**Table 4:** Variables associated with specific patterns of delays compared to "no delays" (multivariable multinominal logistic regression analysis)

	**Pattern 1**	**Pattern 2**	**Pattern 3**	**Pattern 4**	**Pattern 5**
	**Isolated disorders of pronunciation**	**Combined delays of pronunciation, grammar, rhythm of speech and psychosocial development**	**Delays in development of memory, concentration, endurance, abstraction and visual perception**	**Delays of fine body coordination and grapho-motor coordination**	**Combination of delays in cognitive and motor development**
	**OR (95% CI)**	**OR (95% CI)**	**OR (95% CI)**	**OR (95% CI)**	**OR (95% CI)**
Sex					
Male	1.00	1.00	1.00	1.00	1.00
Female	0.50 (0.43-0.58)	0.54 (0.45-0.64)	0.62 (0.55-0.71)	0.21 (0.18-0.25)	0.31 (0.26-0.37)
Nationality					
German	1.00	1.00	1.00	1.00	1.00
Non-German	0.63 (0.47-0.85)	1.32 (0.99-1.75)	2.60 (2.20-3.05)	1.27 (1.02-1.59)	2.06 (1.64-2.60)
Age					
Per year difference	0.82 (0.68-0.99)	0.77 (0.60-0.97)	0.45 (0.38-0.53)	0.26 (0.22-0.32)	0.23 (0.19-0.29)

OR - odds ratio.

CI – confidence interval.

## Discussion

The analysis of data from school-entry examinations in a Lower Bavarian district revealed co-occurrence of delays in closely related development areas in bivariate analysis and a clustering of delays into five distinct patterns associated with sex, age and migration status.

In studies of selected, clinical populations, authors often noted co-occurrence of developmental delays. For example, Kadesjö and Gillberg [25] noted that 6.1% of children in their study population had combined delays in motor body coordination and ability of attention. In several further studies, children with specific developmental disorders were assessed for further impairments [26-30]. Generally, these studies provided evidence for clustering of developmental delays, but their results are not directly comparable with the current study in the normal, unselected population. In addition, all cited studies have in common that they assessed only a narrow selection of developmental delays.

Clustering of multiple developmental delays was not formally investigated yet. Eldred and Darrah [5] used cluster analysis to study developmental delays, but only considering gross motor coordination. In our study, a cluster analysis was carried out with respect to multiple development areas. The patterns we identified are interesting from the point of view of diagnostics on the one side and prevention on the other side. With respect to prevention of negative consequences of the delays, single delays can be addressed by single interventions, while combined delays would require a combination of interventions addressing several aspects at the same time or in sequence. For example, isolated delays in pronunciation can be directly addressed by speech therapy while combined with problems in the use of grammar might require learning the language. The question is interesting –which cannot be answered by the current cross- sectional study- if the delays are independent of each other or if possibly delays in some areas negatively affect developmental chances in other areas: for example, contribute delays in cognitive development to motor development (pattern 5)? In addition, in the patterns 3 and 5 also other developmental delays beyond those used to name the clusters were rather frequent. In such case, particularly these patterns can be seen as complex delays.

Male sex and younger age were consistently identified as being associated with a higher risk of single development delays in previous analyses of the same data [18,19], now they shown to be also associated with combined delays. As for younger age, this is not surprising, since the instrument is assessing the development with respect to abilities required for schooling. Some younger children might not have achieved this developmental stage yet. In such case, there might not be a true developmental delay at individual level, but the assessment is conducted too early. Migration background was less commonly associated with isolated pronunciation problems – likely not because pronunciation problems were less common in the migrants, but rather because in migrants they were more often associated with other delays.

The strengths of the analysis are the large, unselected sample from the normal population, collected from consecutive years and examined by the same medical team. A limitation of our analysis is that only dichotomous outcomes: presence or absence of delays was studied and no information about the severity of delays was collected. A more detailed knowledge of the severity of the delays would allow a better understanding of the need of intervention. Also, we did not study the improvement of delays over time, and it is not fully clear which of those represent just a variation of individual development and which some form of a permanent pathology. In addition, the clinical implications of combined delays are not clear then their long term consequences were not studied yet. We also cannot determine, if interventions would help reducing the burden of delays, but we assume that even if the delays can spontaneously resolve over time, interventions could improve the adjustment of the children.

## Conclusions

Most preschool children are going through an intact development without significant deficits in the acquisition of skills relevant for schooling. However, some children display development delays, and those with delays often have not just a single delay but rather there is some co-occurrence of delays in form of specific patterns. This co-occurrence of delays in multiple areas should be considered in designing intervention strategies as addressing several areas in a parallel fashion might be particularly effective. In the future, more attention should be paid to combined developmental delays, especially regarding combinations of delays of motor function and of cognition. Furthermore, factors associated with specific patterns should be studied more in detail, to identify unfavorable constellations. Also, there is a need to study long term outcomes of children with combined developmental delays in a longitudinal manner. We initiated such study in the region where the reported data was collected.

## Competing interests

The authors declare that they have no competing interests.

## Authors’ contributions

HLS has made substantial contributions to conception and design, has examined the children, analysed and interpreted data and drafted the manuscript. AK has made contributions to the writing of the manuscript. RTM has made contribution to conception and design, supervised the statistical analysis and has been involved in revising the manuscript critically for important intellectual content. All authors have given final approval of the final version of the manuscript.

## Pre-publication history

The pre-publication history for this paper can be accessed here:

http://www.biomedcentral.com/1471-2431/14/18/prepub
